# 90449 Can Ultrasound detect changes to spinal cord blood flow before and after injury?

**DOI:** 10.1017/cts.2021.418

**Published:** 2021-03-30

**Authors:** Amir Manbachi, Nicholas Theodore

**Affiliations:** Johns Hopkins School of Medicine

## Abstract

ABSTRACT IMPACT: To track recovery and mitigate additional spinal cord injury (avoiding further paralysis), we are assessing the applicability of an implantable ultrasound device that can monitor the tissue health postoperatively. OBJECTIVES/GOALS: To date, no method has been developed that monitors spinal cord perfusion rate (mL/min/g) or pressure (mmHg) successfully after the surgery. Our goal is to design, construct, and validate (in animal models) a novel sensor that quantifies postoperative tissue perfusion in patients with SCI at the site of and downstream from the injury. METHODS/STUDY POPULATION: A sample size of 10 animals will allow us to test our hypothesis to track tissue perfusion before and after the SCI using ultrasound. After prepping and scrubbing the animal, the skin will be incised with a blade, bony structures will be removed and the spinal cord will be revealed. A 25-g weight will then be dropped from a height of 15 cm, and the animal will be observed for contraction of the lower extremities, a sign that the cord was damaged. Using Doppler ultrasound settings available on commercial transducers, we will investigate the acceptable frequency, as well as proper Doppler mode with and without contrast agents, and with and without elastography (stiffness mapping of the tissue). A range of frequencies will be tested (5 -25 MHz). RESULTS/ANTICIPATED RESULTS: It is expected that at frequencies 12 MHz and above, our radiologist collaborators would be able to easily detect the blood flow. It is also expected that the injury will have a noticeable effect on the changes of this detected blood flow. We aim to present figures demonstrating ultrasound image qualities obtained at various frequencies. We expect three such figures: one for gray scale ultrasound imaging, one for color Doppler and finally, one for spectral Doppler, which is the one mostly used to quantify blood flow. DISCUSSION/SIGNIFICANCE OF FINDINGS: To monitor recovery and mitigate secondary injury in patients with traumatic SCI, there is a need to monitor tissue perfusion intra-operatively. To address this need, we will design, construct, and validate a novel sensor that will postoperatively quantify tissue perfusion for the SCI patients at the site of the injury.

